# Dual tumor suppressing and promoting function of Notch1 signaling in human prostate cancer

**DOI:** 10.18632/oncotarget.10333

**Published:** 2016-06-30

**Authors:** Karine Lefort, Gian Paola Ostano, Maurizia Mello-Grand, Valérie Calpini, Maria Scatolini, Antonella Farsetti, Gian Paolo Dotto, Giovanna Chiorino

**Affiliations:** ^1^ Department of Biochemistry, University of Lausanne, Lausanne, Switzerland; ^2^ Laboratory of Cancer Genomics, Fondazione “Edo ed Elvo Tempia Valenta”, Biella, Italy; ^3^ Laboratory of Molecular Oncology, Fondazione “Edo ed Elvo Tempia Valenta”, Biella, Italy; ^4^ Institute of Cell Biology and Neurobiology, National Research Council, Rome, Italy; ^5^ Internal Medicine Clinic III, Goethe University, Frankfurt am Main, Germany; ^6^ Cutaneous Biology Research Center, Massachusetts General Hospital, Charlestown, MA, USA

**Keywords:** Notch, prostate cancer

## Abstract

Adenocarcinomas of the prostate arise as multifocal heterogeneous lesions as the likely result of genetic and epigenetic alterations and deranged cell-cell communication. Notch signaling is an important form of intercellular communication with a role in growth/differentiation control and tumorigenesis. Contrasting reports exist in the literature on the role of this pathway in prostate cancer (PCa) development. We show here that i) compared to normal prostate tissue, Notch1 expression is significantly reduced in a substantial fraction of human PCas while it is unaffected or even increased in others; ii) acute Notch activation both inhibits and induces process networks associated with prostatic neoplasms; iii) down-modulation of Notch1 expression and activity in immortalized normal prostate epithelial cells increases their proliferation potential, while increased Notch1 activity in PCa cells suppresses growth and tumorigenicity through a Smad3-dependent mechanism involving p21^WAF1/CIP1^; iv) prostate cancer cells resistant to Notch growth inhibitory effects retain Notch1-induced upregulation of pro-oncogenic genes, like EPAS1 and CXCL6, also overexpressed in human PCas with high Notch1 levels. Taken together, these results reconcile conflicting data on the role of Notch1 in prostate cancer.

## INTRODUCTION

Prostate cancer (PCa) is the most commonly diagnosed malignancy in men, as a result of population aging [1] and PSA testing, which has favored early detection but also overdiagnosis and overtreatment.

Unlike the well-characterized pathway of acquired mutations displayed by colon cancer, there is not a clearly defined unique pathway for the development and progression of malignant disease in the prostate [2, 3]. A distinguishing feature is the heterogeneous and multifocal nature of PCa, with a juxtaposition of hyperplastic and preneoplastic (PIN) lesions with neoplastic foci of various degree of malignant progression. This suggests that PCa development is the combined result of genetic and epigenetic alterations in the cells of origin and changes in their communication with their environment [4–7].

The Notch signaling pathway is an important form of cell-cell communication that plays a key role in control of cell fate, stem cell potential and differentiation [8]. The Notch gene family (Notch1, 2, 3, 4) consists of evolutionary conserved transmembrane receptors. Interactions with ligands of the Delta and Jagged families present on the surface of neighboring cells trigger proteolytic cleavage of these receptors with release of their intracellular region. The best characterized “canonical” pathway of Notch activation involves translocation of this molecule to the nucleus, where it associates with the DNA binding protein CSL (CBF-1 or RBP-Jκ), converting it from a repressor into an activator of transcription [8, 9]. The biological function of the Notch pathway is highly context-dependent, as it can exert opposite roles in growth/differentiation of different cell types, at different developmental stages and/or under normal vs pathological conditions. In mammalian cells, increased Notch signaling has been previously implicated as a positive determinant of tumorigenesis, which is associated with the ability of this pathway to suppress differentiation [10–16]. However, in keratinocytes of mouse and human origin, Notch signaling plays the opposite function of promoting differentiation and suppressing tumor formation [17–19]. Prostate epithelial cells share some significant similarities with keratinocytes, including their distribution in stem cell and transit amplifying compartments, and cells at various stages of differentiation [20, 21]. In mouse prostate, inactivation of the Notch pathway disrupts normal commitment to differentiation and causes enhanced proliferation with features similar to early preneoplastic lesions [22]. Conflicting reports exist on a possible tumor suppressing or promoting function of Notch in (human) prostate cancer: several works suggest that Notch1 may act as a tumor suppressor in this specific tissue and its signalling is lost in prostate adenocarcinomas [22, 23]; other surveys have demonstrated that Notch1 expression augments in human prostate tumor specimens with increasing tumor grade and that Notch1 knockdown decreases cell invasion [24, 25]. Thus, an important question is whether these conflicting conclusions can be, at least to some extent, reconciled. A recent review by Carvalho and coworkers reveals that increased Notch1 can confer a survival advantage on prostate cancer cells, but also that Notch1 signaling can antagonize growth and survival of both benign and malignant prostate cells [26]. In another review, Su and colleagues stress the need of future comprehensive studies to clarify the role of Notch signaling in PCa, which remains inconclusive until now [27].

Determining the cellular contexts where Notch1 promotes or suppresses prostate growth could open opportunities for diagnostic and therapeutic interventions. This is an important and urgent need, in view of the proposed use of anti-Notch compounds for cancer therapy, including prostate cancer [28]. We show here that, while increased Notch1 exerts a growth suppressing function in both human prostate primary epithelial cells and cancer cells, at the gene expression level it induces genes with opposite functions. One group of genes can contribute to growth/tumor suppression, while a second group of genes has an opposite growth-promoting function, that can be unmasked in cells that become resistant to the Notch-growth inhibitory effects.

## RESULTS

### Notch1 expression and activity are consistently down-modulated in prostate cancer cell lines and a substantial fraction of prostate cancers but up-regulated in others

Interrogating public data repositories (i.e. Oncomine, GEO, ArrayExpress and the cBioportal), we found conflicting information on levels of Notch1 mRNA in prostate cancer. In some datasets Notch1 expression was found significantly down-regulated [29–34] and (TCGA PRAD (Prostate Adenocarcinoma)), while in others it did not appear to change [35, 36] or even to increase [37].

**Figure 1:**
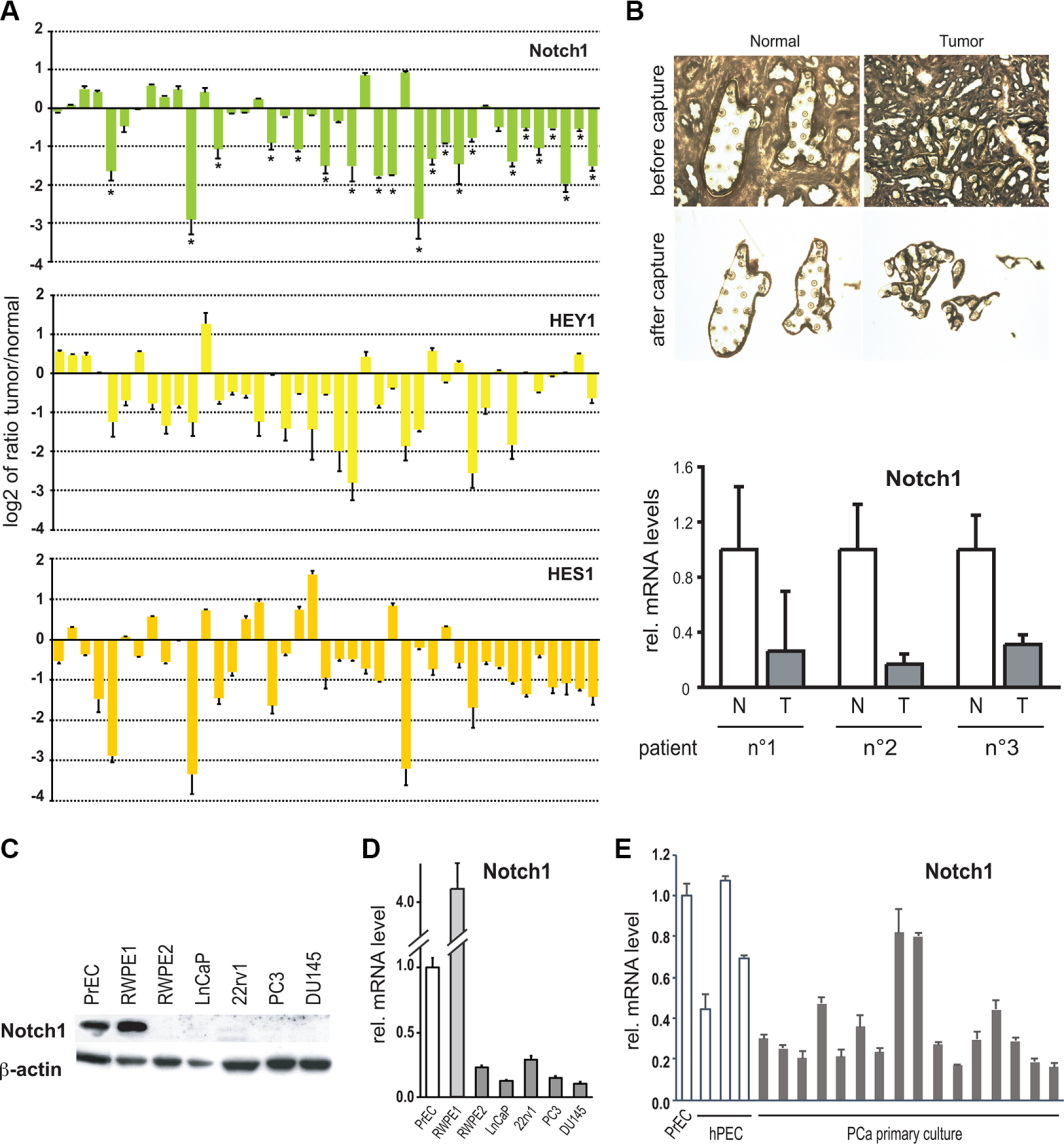
Notch1 expression and activity are down-modulated in human prostate tumors and cancer cells lines (**A**) Total RNA from 41 surgically excised prostate carcinomas were analyzed in parallel with 10 normal prostates and/or a commercially available pool of RNA extracted from organ donor healthy prostates for levels of Notch1, HEY1 and HES1 mRNA expression by real time RT-PCR. Results are expressed as log2 ratios of mRNA levels in the individual tumors versus the averaged values of normal prostate samples (using for each samples β-actin for internal normalization). Differences in gene expression below −0.5 are all statistically significant, as calculated by two sample unpaired Student *t*-test. (**B**) LCM-obtained epithelial cells from prostate tumor (T, *n* = 3) and normal prostate tissue (N, *n*= 3) samples from different individuals were analyzed by RT–qPCR for the Notch1 gene. (**C**) Human primary prostate epithelial cells (PrEC), immortalized prostate epithelial cells (RWPE1), a *ras*-induced tumorigenic derivative of RWPE1 cells (RWPE2; [38]), and the indicated prostate cancer cell lines (LnCaP, 22rv1, PC3 and DU145) were analyzed by immunoblotting for the Notch1 protein with β-actin as equal loading control. (**D** and **E**) The same set of cell lines as in the previous panel as well as other human primary prostate epithelial cells (hPEC) and immortalized prostate epithelial cancer cells (C1 IM to C45 IM) were analyzed for levels of Notch1 mRNA by real time RT-qPCR. Values are in relative arbitrary units after 18s or β-actin normalization.

We directly evaluated Notch1 mRNA levels in 41 prostate adenocarcinomas and 10 age-matched controls (non tumor prostate tissues derived from the peripheral zone of the prostate gland) and in 3 independent prostate epithelial tumors versus normal matched tissues after laser capture microdissection (LCM), by RT-qPCR analysis. Interestingly, even though Notch1 expression levels were in average decreased in tumor samples when compared to normal ones, including LCM samples, some PCa samples showed low levels, while others unchanged or increased levels when compared to normal prostate tissues (Figure 1A, 1B). This pattern of different Notch expression levels was also observed in Tomlins' LCM dataset [31], assuring no bias due to stroma or non neoplastic epithelial tissue.

Decreased levels of Notch1 protein and mRNA expression were also found in several widely used prostate cancer cell lines (RWPE2, LNCaP, 22rv1, PC3 and DU145) relatively to primary prostate epithelial cells (PrEC or hPEC) or to an immortalized but non-tumorigenic prostate cell line (RWPE1) [38] (Figure 1C–1D). A majority of other prostate cell lines derived from a panel of benign and malignant disease also showed lower Notch1 levels relative to commercially available primary prostate epithelial cells (PrEC), as well as 3 other independently derived primary prostate epithelial cells (Figure 1E). There were no significant differences in Notch2 expression in the human tumors that we analyzed and levels of Notch2 mRNA were only slightly down-modulated in prostate cancer cell lines (data not shown).

Expression levels of “canonical” Notch target genes of the Hes/Herp family are commonly assessed as an indication of endogenous Notch activity. When assessed by RT-qPCR, Hes1 and Hey1 were found significantly decreased in approximately 70% of the prostate tumors that we examined (Figure 1A). However, there was only partial concordance between tumors with decreased Notch1 versus Hes1 or Hey1 levels (66% and 83% concordance, respectively). Co-occurrence analysis using the largest dataset available (TCGA-PRAD), yielded that Notch1 association with Hey1 is statistically significant (*p*-value = 0.004), as well as Notch1 association with Notch3 (*p*-value = 0.008), Notch4 (*p*-value = 0.0009) and Jag2 (*p*-value = 0.00001). With Notch2 and Jag1, there is a tendency towards co-occurrence, however the *p*-value is greater than 0.01 (0.02 for Notch2 and 0.28 for Jag1).

### Notch1 related expression profiling of clinical tumors reveals a dual pattern of gene expression

**Figure 2:**
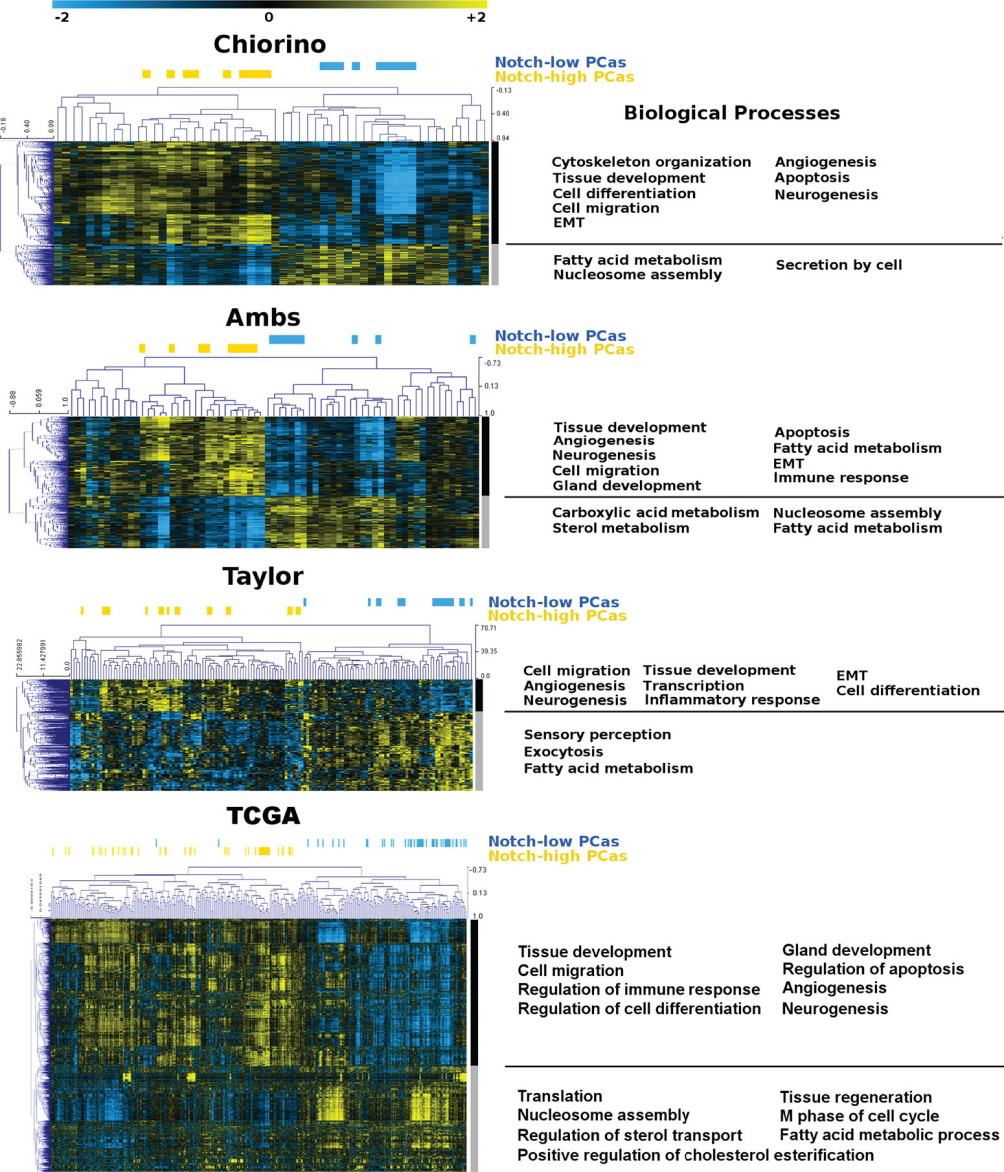
Heatmap of prostate tumors and Notch1-related genes Our own gene expression dataset of prostate cancer, named Chiorino and three independent ones ([36, 33], TCGA). were analyzed and tumors classified into Notch_low or Notch_high, according to Notch1 expression levels in tumors versus healthy controls (blue and yellow horizontal bars, respectively). Unsupervised hierarchical clustering and functional enrichment analysis was performed on genes over-expressed in Notch_high versus Notch_low tumors (black vertical bars) and on genes over-expressed in Notch_low versus Notch_high (grey vertical bars).

We compared expression profiles of PCa lesions with low versus high levels of Notch1, using our own (Chiorino) and 3 independent datasets [33, 36] (TCGA-PRAD). GO analysis revealed an astonishingly similar pattern of affected biological processes, among the 4 independent datasets. Genes up-regulated in tumors with Notch1 levels higher than normal tissue (Notch_high) were enriched in terms like cell migration, apoptosis, angiogenesis, neurogenesis and epithelial to mesenchymal transition, while fatty acid metabolism and nucleosome assembly were consistently overrepresented among the genes up-regulated in PCas with Notch1 levels lower than normal prostate tissue (Notch_low) in the 4 datasets analyzed (Figure 2).

We then analyzed the global expression changes of LNCaP and PC3 cells in response to acute Notch activation by gene profiling with oligo microarrays using the Affymetrix platform. Gene network analysis with Metacore software revealed that 57% of the network objects induced by Notch activation in PC3 and/or LNCaP cells (559/982) and 54% of those suppressed by Notch activation (460/849) are involved in prostatic neoplasms. Furthermore, we found a significant enrichment of process networks associated with prostatic neoplasms (Table 1), both for induced (Regulation of progression through cell cycle, Cell proliferation) and for suppressed genes (Inflammatory response, Regulation of progression through cell cycle, Cell proliferation).

**Table 1:** Gene network analysis

Networks	*p* Value	#	Network Objects
Prostatic Neoplasms Inflammatory response	9.151E-03	6	C/EBP, Thrombospondin 1, TGF-beta2, AP-1, Galpha(q)-specific peptide GPCRs, IL1RN
Prostatic Neoplasms Regulation of progression through cell cycle	1.445E-02	11	PPAP, BIN1 (Amphiphysin II), Cyclin D2, TGF-beta2, c-Myc, AP-1, c-Jun/c-Jun, VEGF-C, c-Jun, PKC, Cyclin D
Prostatic Neoplasms Cell proliferation	2.465E-02	7	BIN1 (Amphiphysin II), Androgen receptor, TGF-beta2, c-Myc, PKC-mu, PKC, Ebp1
Network analysis on genes down-regulated by Notch1 activation in LNCaP and/or PC3 cell lines.			

Networks	*p* Value	#	Network Objects
Prostatic Neoplasms Regulation of progression through cell cycle	1.971E-08	26	p21, Casein kinase II, alpha chains, K-RAS, PCNA, IGF-1 receptor, TGF-beta 1/3, Tcf(Lef), Cyclin A, Skp2/TrCP/FBXW, PLK1, Cyclin D1, CDC25, N-Ras, c-Raf-1, b-Myb, CDC20, SKP2, RAD9, CDC25B, STAT1, PKC, Brca2, CDK1 (p34), FGF1, Casein kinase II
Prostatic Neoplasms Cell proliferation	1.521E-04	14	CDC25C, PCNA, TGF-beta1/3, Skp2/TrCP/FBXW, PLK1, CDC25, Amphiregulin, c-Raf-1, Pim-1, CDC25A, SKP2, PKC, FGF1

Network analysis on genes up-regulated by Notch1 activation in LNCaP and/or PC3 cell lines.

Taken together, these results are consistent with a putative dual role of Notch1 gene in prostate cancer, where it may act both as a tumor suppressor and as an oncogene.

### Counteracting effects of Notch1 expression on prostate epithelial cell growth and tumorigenesis

To demonstrate that Notch1 can act as a tumor suppressor in prostate cancer, we assessed the consequences of i) decreased Notch1 expression on proliferation of non-tumorigenic prostate epithelial cells and ii) increased Notch1 expression in prostate cancer cells with low Notch1 levels.

**Figure 3:**
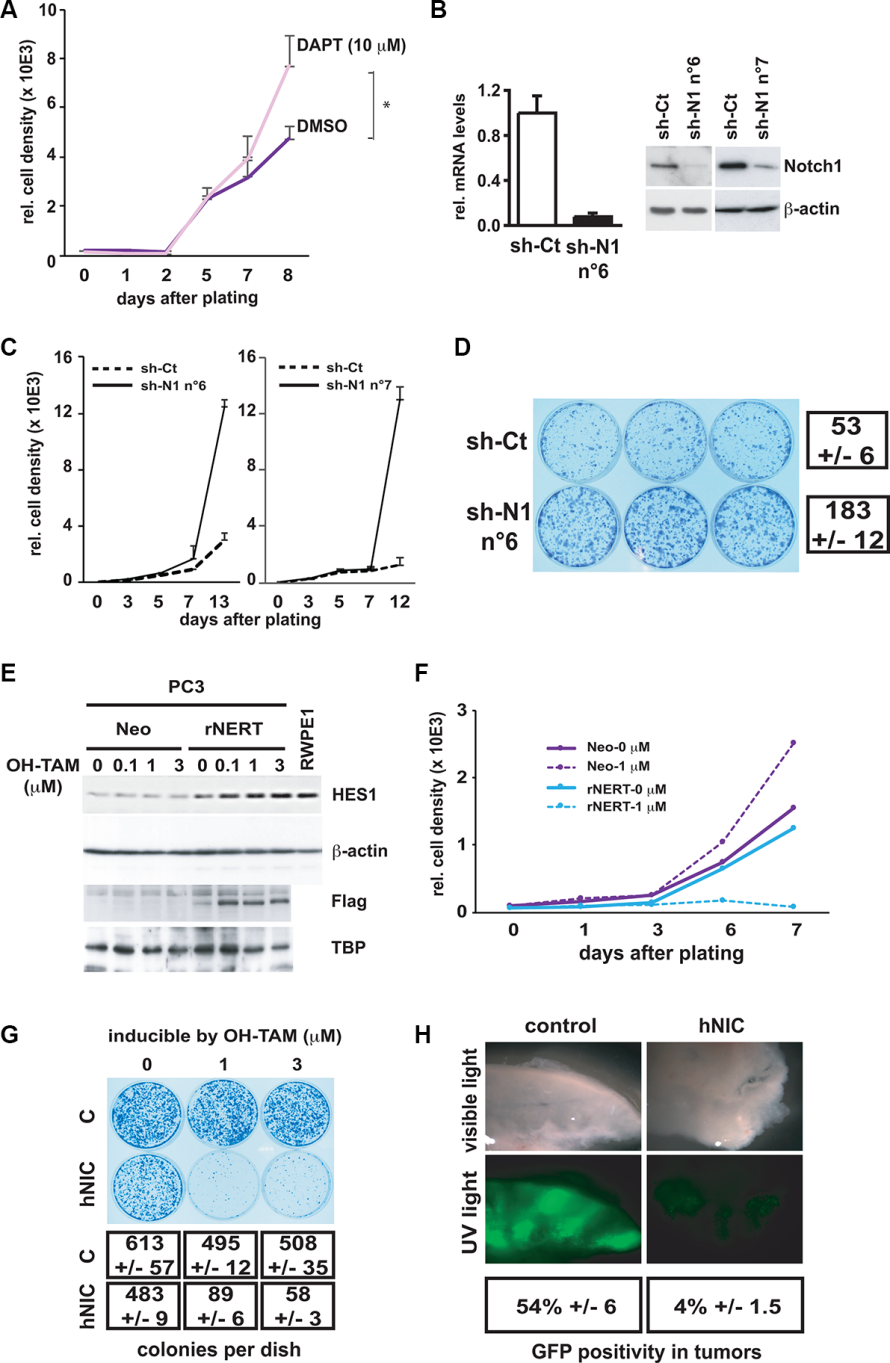
Inhibition of Notch1 expression in normal prostate epithelial cells enhances cell growth whereas activated Notch1 expression in prostate cancer cells inhibits growth and tumorigenesis (**A**) Immortalized prostate epithelial cells (RWPE1) were incubated with the γ-secretase inhibitor DAPT at 10 μM or with DMSO at day 0 and cell proliferation was assayed everyday by Alamar Blue assay until day 8. *p < 0.05 (paired student *t*-test). (**B**) RWPE1 cells were stably infected with a retrovirus expressing an shRNA against Notch1 (sh-N1 n°6 and n°7) or empty vector control (sh-Ct). Notch1 mRNA and protein levels were determined by real time RT-qPCR (with 36β4 mRNA for normalization) and immuno-blotting. (**C** and **D**) The same stably infected RWPE1 cells were examined for their proliferation potential using the Alamar Blue growth/density assay (plating 200 cells per well of a 96 well plate in triplicate) (C) or clonogenicity (plating 2000 cells per 6 cm plate in triplicate; staining of dishes 13 days after plating) (D). (**E**) Prostate cancer cells (PC3) were stably infected with a retroviral vector expressing a flag tagged-activated Notch1 protein fused to the human estrogen receptor (rNERT), or empty vector control (Neo). Cells were subsequently treated with 4-hydroxytamoxifen (OH-TAM) at the indicated concentrations and collected 3 days later. Nuclear extracts were analyzed by immunoblotting with anti-flag antibodies for detection of the rNERT protein, which is not recognized by antibodies against endogenous Notch1. Immuno-blotting for TBP was used for equal loading control. Total extracts of the same cells were analyzed by immunoblotting for HES1 protein levels, in parallel with RWPE1 cells, using β-actin as equal loading control. (**F** and **G**) Proliferation of the same PC3 cells as in the previous panels was assessed by Alamar Blue growth (F) or clonogenicity (G) assays plus/minus treatment with OH-TAM at the indicated concentrations. Numbers of colonies represent the average of triplicate dishes. (**H**) PC3 cells were infected with retroviruses expressing the activated Notch1 together with GFP or GFP alone as control, mixed with uninfected PC3 cells in a ratio 1:3 and injected subcutaneously (in matrigel; 10^6^ cells per injection) into nude mice. Four weeks later, tumors were dissected, sectioned and visualized directly by phase contrast and fluorescent microscopy under a dissecting microscope. Quantification of GFP positive areas was obtained using the ImageJ software (http://rsb.info.nih.gov/ij) for digitally acquired images of 3 different tumors for each combination of cells. Values are expressed as average percentage area of GFP positivity versus total tumor area.

First, immortalized RWPE1 prostate epithelial cells, which express Notch1 at high levels, were incubated with the γ-secretase inhibitor DAPT to inhibit Notch signaling, and this had a positive effect on proliferation by day 10 of treatment (Figure 3A). The same cells were infected with two retroviruses expressing different shRNAs against Notch1 (sh-N1 n°6 and n°7) in parallel with an empty vector control. Effective down-modulation of the Notch1 gene also in this case caused a substantial increase of proliferative potential, as assessed by Alamar Blue cell proliferation as well as clonogenicity assays (Figure 3B–3D).

Conversely, PC3 cells which express Notch1 at low levels were stably infected with a retrovirus expressing the constitutively active form of Notch1 fused to the estrogen receptor (rNERT) [39]. Cells were treated with 4-hydroxytamoxifen at various concentrations to induce translocation of the Flag tagged-Notch fusion protein to the nucleus. This caused a parallel increase of the Notch target protein HES1, to levels similar to those found in RWPE1 cells (Figure 3E). The same treatment caused drastic growth inhibition of the rNERT expressing cells before day 6, as assessed by both Alamar Blue and clonogenicity assays (Figure 3F, 3G). We note that even without tamoxifen treatment, rNERT expressing cells exhibited lower proliferation than the controls, consistent with some “leakage” of the Notch fusion protein to the nucleus, and consequent effects on downstream HES1 gene expression. In parallel experiments, PC3 cells were infected with a retrovirus expressing activated Notch1 (hNIC) together with green fluorescent protein (GFP) or with a control retrovirus expressing GFP alone. The initial fraction of infected cells (% of GFP-positive cells) was similar with the two viruses. However, already 3 days after cell transduction and by 9 days of cultivation, there was a progressive decrease in the fraction of cells infected with the Notch-GFP virus, while the fraction of cells infected with the GFP virus remained constant (Supplementary Figure S1A).

To assess whether increased Notch1 activity results also in a tumorigenic growth disadvantage *in vivo*, mice were injected subcutaneously with an admixture of PC3 cells infected with either the activated Notch1 and GFP expressing retrovirus, or the virus expressing GFP only, admixed with uninfected cells. The tumors formed by a mixture of cells infected with the control GFP virus and uninfected cells were composed of several large areas of GFP-positive cells, distinct from neighboring areas composed of GFP-negative cells (Figure 3H). This pattern of growth is in agreement with the finding within established cancer cell lines, including PC3, of distinct sub-populations with different growth and tumorigenic potential [40]. When similar experiments were repeated with a mixture of cells infected with a retrovirus expressing activated-Notch1 together with GFP, little or no GFP-positive tumor areas were detected, consistent with the long term growth suppressing effects of activated Notch1 in culture (Figure 3H).

### Notch growth suppressing effects in prostate cancer cells are mediated by p21^WAF1/CIP1^ in a Smad3 dependent manner

**Figure 4:**
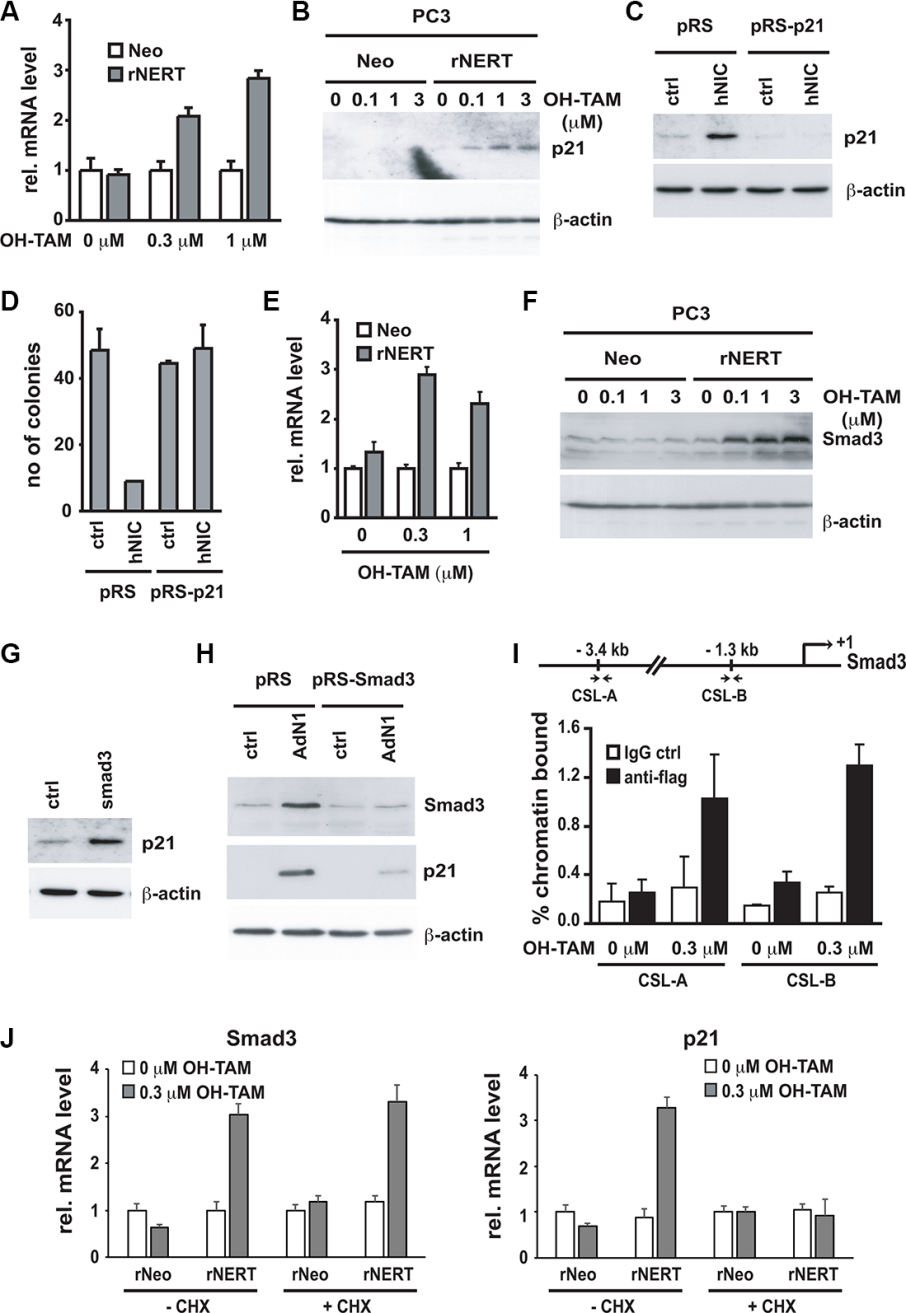
p21^WAF1/Cip1^ mediates Notch1-induced growth arrest and is controlled by Notch1 through a Smad3 dependent mechanism (**A** and **B**) PC3 cells stably infected with a retroviral vector expressing the flag tagged-activated Notch1 protein fused to the human estrogen receptor (rNERT), or control virus (Neo) were treated for 3 days with 4-hydroxytamoxifen at the indicated concentrations. Expression of p21^WAF1/Cip1^ was analyzed by real time RT-PCR (using 36β4 mRNA for normalization) (A) and by immuno-blotting (B). (**C**) PC3 cells were stably infected with a retrovirus expressing an shRNA against p21^WAF1/Cip1^ (pRS-p21 n°1) or empty vector control (pRS). Cells were subsequently transfected with an expression vector for activated Notch1 (hNIC) together with a neomycin resistance gene, or empty vector control (ctrl). Twenty four hours later, cells were analyzed by immuno-blotting for p21^WAF1/Cip1^ expression, with β-actin for normalization. (**D**) PC3 cells plus/minus p21^WAF1/Cip1^ knock-down and activated Notch1 expression as in the previous panel were replated (at 8 hours after transfection) at low density in triplicate dishes (1000 cells per 6 cm dish) in the presence of neomycin for selection of transfected cells. Numbers of colonies per dish were counted 10 days later. (**E** and **F**) The same PC3 cells with inducible Notch1 activity as in (A and B) were analyzed for Smad3 expression by real time RT-PCR (using 36β4 mRNA for normalization) (E) and immunoblotting (F). (**G**) PC3 cells were transiently transfected with an expression vector for Smad3 (Smad3) or empty vector control (ctrl) for 48 hours, followed by immunoblot analysis of p21^WAF1/Cip1^ and β-actin proteins. (**H**) PC3 cells were stably infected with a retrovirus expressing shRNA against Smad3 (pRS-Smad3 n°1) or empty vector control (pRS) and subsequently infected with an activated Notch1 expressing adenovirus (AdN1) or GFP-expressing control (ctrl). Twenty four hours later, cells were analyzed by immunoblotting for levels of Smad3 and p21^WAF1/Cip1^ proteins with β-actin for normalization. (**I**) The same PC3 cells with inducible Notch1 activity as in (A and B) were processed for chromatin immunoprecipitation with antibodies specific for flag or non-immune IgG control followed by PCR amplification (50 cycles) of various regions of the Smad3 promoter as indicated in the schematic above. The sequences of the two predicted RBP-binding sites (CSL-A and CSL-B) are, respectively, 5′-CTAATGGGAAAATAA-3′ and 5′-GGGGTGGGAGATTCC-3′. Un-precipitated chromatin preparations were similarly analysed and used as “input DNA” control. Binding of the rNERT-flag fusion protein to its two predicted CSL binding sites in the Smad3 promoter was quantified by chromatin immuno-precipitation assay and real time PCR. The amount of precipitated DNA was calculated relative to the total input chromatin, and expressed as percentage of the total according to the following formula (Frank et al. 2001) : % total = 2^ΔCt^x5 where ΔCt = Ct (input)-Ct (immuno-precipitation). Ct: cycle threshold. (**J**) PC3 cells expressing the rNERT fusion protein were treated, in parallel with control cells (Neo), for 24 hours with 4-hydroxytamoxifen in presence or absence of cycloheximide (CHX, 10 mg/ml, added 2 hours before 4-hydroxytamoxifen treatment). Expression of Smad3 (left panel) and p21^WAF1/Cip1^ (right panel) was analyzed by real time RT-PCR (using 36β4 mRNA for normalization).

To better investigate the growth suppressing effects of Notch1 in prostate cancer cells expressing low levels of this gene, we looked for well-established tumor suppressors among the genes induced by Notch1 in PC3 cells. As shown in Table 1, tumor suppressor p21^WAF1/CIP1^ contributes to the overrepresentation of “regulation of progression through the cell cycle in prostatic neoplasms” within the list of genes up-regulated by Notch1. A number of studies have shown that expression of p21^WAF1/CIP1^ is reduced in human prostate cancer [41, 42]. Its expression strongly increases upon Notch1 upregulation (FC = 3.3 and adjusted *p*-value = 0.0003 in PC3 cells by microarray data, Supplementary Table 1). Expression of activated Notch1 (via a construct encoding Notch1-ER fusion protein) in PC3 cells followed by RT-qPCR analysis confirmed a significant increase of p21^WAF1/CIP1^ levels already at low levels of activated Notch1, sufficient for growth suppression (Figure 4A, 4B). To assess the functional significance of these findings, PC3 cells were infected with retroviruses expressing two different shRNAs against p21^WAF1/CIP1^ in parallel with an empty vector control (pRS) (Figure 4C). Cells were subsequently transfected with a plasmid expressing activated Notch1 together with a selectable marker. Activated Notch1 expression caused significant growth suppression, as assessed by colony formation, of control cells but not of PC3 cells with p21^WAF1/CIP1^ knock-down (Figure 4D).

p21^WAF1/CIP1^ expression is controlled by multiple convergent mechanisms [43]. While p53 is one of its key positive regulators, the p53 protein was undetectable in PC3 cells (data not shown), consistent with the previously reported deletion of the p53 gene in these cells [44]. We have previously shown that p63, another p53 family member can regulate p21^WAF1/CIP1^ expression in keratinocytes [45]. However, in PC3 cells, p63 silencing did not have positive effects on p21^WAF1/CIP1^ expression (Supplementary Figure S1B). Another positive regulator of p21^WAF1/CIP1^ expression is Smad3, which was also previously reported to be downmodulated in prostate cancer [30, 31, 35, 46–51]. As with p21^WAF1/CIP1^, Smad3 levels were up-regulated in PC3 cells by activated Notch1 expression (FC = 2.9 and adjusted *p*-value = 0.001 in PC3 cells by microarray data, Supplementary Table 1). Using the inducible Notch1 expression system, Smad3 mRNA and protein expression were found to increase in PC3 cells in a dose-dependent manner paralleling induction of p21^WAF1/CIP1^ (Figure 4E, 4F). Transfection of these cells with a Smad3 expression vector caused induction of p21^WAF1/CIP1^ expression (Figure 4G) while, conversely, knock-down of Smad3 by infection with shRNA retroviruses (pRS-Smad3 n°1 and n°2) showed that induction of p21^WAF1/CIP1^ by activated Notch1 is Smad3-dependent (Figure 4H).

Nucleotide sequence analysis revealed the presence of numerous fully conserved CSL binding sites dispersed throughout the promoter and the large (> 100 kb) first intronic region of the Smad3 gene to which the rNERT fusion protein was found to bind in a 4-hydroxytamoxifen dependent manner (Figure 4I). To assess whether this gene is induced as a primary consequence of increased Notch1 signaling and consequent activation of the transcription factor CSL, PC3 cells expressing the rNERT fusion protein were treated with 4-hydroxytamoxifen only for 24 hours, at a time when p21^WAF1/CIP1^ expression was not yet induced (data not shown). As shown in Figure 4J, unlike p21, Smad3 transcription was already induced even when new protein synthesis was inhibited by cycloheximide prior treatment (Figure 4J).

To confirm our findings in the clinical setting, we analyzed Notch1, p21^WAF1/CIP1^ and smad3 co-occurrence in the TCGA-PRAD dataset and found that Notch1 association with Smad3 was positive and statistically significant (*p*-value = 0.002) and that Notch1 showed a tendency towards co-occurrence with p21^WAF1/CIP1^ (*p*-value = 0.04).

Taken together, these data indicate that Notch growth suppressing effects in prostate cancer cells with low Notch1 levels are mediated by p21^WAF1/CIP1^ in a Smad3 dependent manner.

### Notch-dependent expression of pro-oncogenic genes in prostate cancer cell lines resistant to Notch growth inhibition

As previously said, several published prostate cancer datasets contain samples with upregulated Notch1 expression compared to normal prostate tissue. Furthermore, we showed in 4 independent prostate cancer patient cohorts that tumors with high Notch1 expression are enriched in GO terms as neurogenesis, response to estrogen stimulus and cell migration. To better understand the putative tumor promoting role of Notch1 in these prostate samples, we crossed Notch1-induced or repressed genes in PC3 and/or LNCaP cells with the genes differentially expressed in tumors according to Notch1 levels in at least 3 independent datasets (Supplementary Table 2). Indeed, among the genes induced by Notch1 in cells and up-regulated in tumors with high Notch1 levels, besides the Notch1 target HES1, we found many genes with tumor-promoting functions. Conversely, among the genes inhibited by Notch1 in cells and down-regulated in Notch_high PCas there were targets with tumor suppressing function in prostate cancer (Table 2).

**Table 2:** List of tumor promoting/suppressing genes that are induced/repressed by activated Notch1 in PC3 and or LNCaP cells and concomitantly differentially expressed in prostate tumors according to Notch1 levels

Tumor promoting genes up-regulated in Notch-high PCas and induced by Notch1		
Symbol	Gene Name	Reference
HES1	hes family bHLH transcription factor 1	PMID: 24684754
EPAS1	endothelial PAS domain protein 1	PMID: 21372204
ATP1B1	ATPase, Na+/K+ transporting, beta 1 polypeptide	PMID: 21919029
ZFP36L1	ZFP36 ring finger protein-like 1	PMID: 20802528
SNAI2	snail family zinc finger 2	PMID: 20506051
OAT	ornithine aminotransferase	PMID: 18202758
MAP3K8	mitogen-activated protein kinase kinase kinase 8	PMID: 21267413
CXCL6	chemokine (C-X-C motif) ligand 6	PMID: 23536554
ID1	inhibitor of DNA binding 1, dominant negative helix-loop-helix protein	PMID: 23342268 PMID: 17177845 PMID: 15041724 PMID: 15905202 PMID: 12016143

Tumor suppressing genes down-regulated in Notch-high PCas and inhibited by Notch1		
Symbol	Gene Name	Reference
SLC27A2	solute carrier family 27 (fatty acid transporter), member 2	PMID: 19781100
ENDOD1	endonuclease domain containing 1	PMID: 21829708
CCNG2	cyclin G2	PMID: 24293374
NEDD4L	neural precursor cell expressed, developmentally down-regulated 4-like, E3 ubiquitin protein ligase	PMID: 19004604 PMID: 14615060

**Figure 5:**
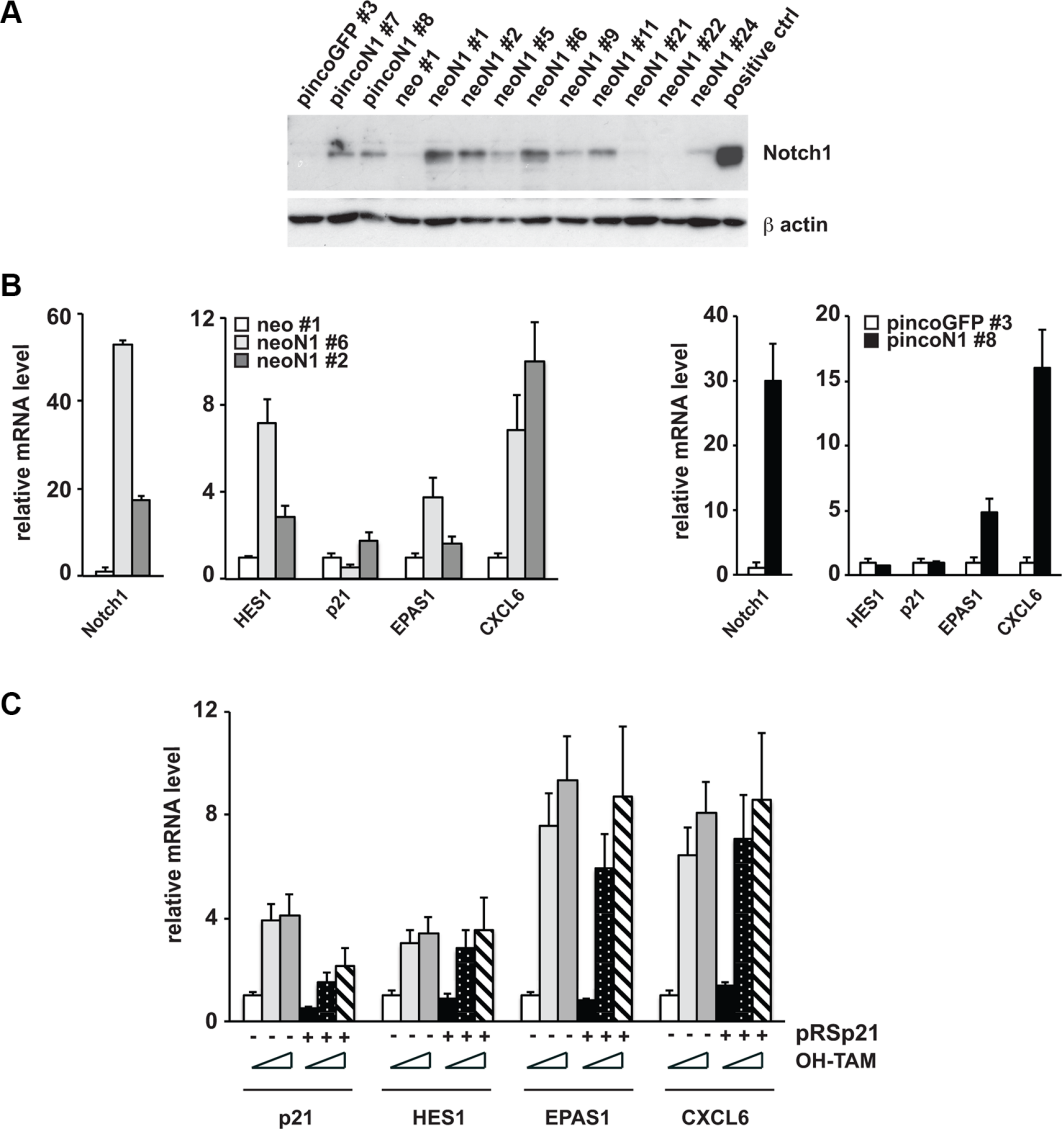
Prostate cancer cell lines resistant to Notch growth inhibition overexpress the oncogenic genes EPAS1 and CXCL6 regardless of p21^WAF1/cip1^ expression levels (**A** and **B**) PC3 prostate cancer cells stably transduced with MSCV-neo-Notch1(neoN1) or pinco-Notch1 (pincoN1) versus respective controls were grown at low density and after 10 days, resistant clones were collected individually, expanded and analyzed by immuno-blotting for Notch1 expression (A) or by RT-qPCR for expression of the indicated genes (B). (**C**) PC3 cells stably transduced with the rNERT versus neo control were subsequently stably infected with a retrovirus expressing an shRNA against p21^WAF1/Cip1^ (pRS-p21, +) or empty vector control (pRS, −) and treated with 4-hydroxytamoxifen (OH-TAM) at 0, 0.3 and 1 μM and collected 2 days later. Expression of the indicated genes was analyzed by RT-qPCR with 36β4 for normalization.

An attractive possibility suggested by the above findings is that there may be an *in vivo* selection for prostate cancer cells resistant to Notch growth inhibitory effects that have retained Notch-induced up-regulation of pro-oncogenic genes. We reasoned that a similar situation may be reproduced and tested *in vitro*. PC3 cells were stably transduced with activated Notch1 retroviruses in parallel with controls and cells were selected. Out of the majority of prostate cancer cells that are growth arrested by activated Notch1 expression, we were able to select a few colonies that kept proliferating. As shown in Figure 5A, some such clones effectively lost Notch1 expression while some retained high Notch1 protein levels. For subsequent analysis, we chose 2 Notch1 “resistant” clones arising from stably infected PC3 cells with MSCV-Notch1 and subsequently selected with neomycin and 1 arising from stably infected PC3 cells with pinco-Notch1-GFP selected for GFP expression. RT-qPCR analysis of CXCL6 and EPAS1, two tumor promoting genes significantly more expressed in human prostate cancer versus normal tissue [52, 53] and induced by Notch1 in LNCaP and PC3 cells (Supplementary Table 2), were also found highly transcribed in the Notch1 resistant clones, like in the *in vivo* tumors with elevated Notch expression (Figure 5B). Furthermore, expression levels of these genes were not affected by knocking down p21^WAF1/CIP1^ in PC3 cells, indicating that while certain tumor suppressor genes are under p21^WAF1/CIP^1 control, the pro-oncogenic Notch target genes might escape from this control (Figure 5C). This possibility deserves further investigation to functionally describe a mechanism.

## DISCUSSION

Among the distinguishing features of prostate tumors are their slow development and multi-focality that point to an interplay between cells of origin, genetic and epigenetic alterations in the developing cancer cell population and changes in the surrounding environment [2]. Mechanisms underlying the balance between growth and differentiation of tumor stem cells are likely to depend, at least in part, on developmental pathways functional also in normal tissues. We addressed this question as it relates to the role of Notch signaling in this context.

In contrast to the tumor promoting function commonly attributed to this pathway in mammalian cells, our prior work demonstrated that Notch signaling contributed in suppression of mouse and human keratinocyte tumor development, by essentially affecting global control of gene expression and differentiation [18, 19, 54]. As for prostate cancer, contrasting reports exist [22–26, 55]. Our present findings indicate that Notch signaling appears to exert a similarly important tumor growth and suppressing function in the prostate. More specifically, we found that Notch1 overexpression in prostate cancer cell lines both induces and inhibits gene networks associated with cell cycle and proliferation in prostatic neoplasms. Furthermore, by comparing several PCa datasets with a focus on Notch1 expression in tumor *vs* normal prostate tissue, we found that the majority of samples was expressing low Notch1 levels. On the contrary, some others were displaying higher levels as compared to the normal counterpart. Bioinformatic comparison between Notch1_low and Notch1_high prostate tumors in four independent datasets allowed characterization of the gene networks overrepresented in the two subgroups: nucleosome assembly and fatty acid metabolism in Notch1_low tumors and EMT, cell migration, angiogenesis and neurogenesis in Notch_high tumors, respectively. Then, in normal prostate epithelial cells endogenously expressing Notch1 at high levels, consequences of knock down resulted in enhanced cell growth, while induction of activated Notch1 in prostate carcinoma cells derived from Notch1_low tumors caused cell growth inhibition and suppressed tumorigenicity. Accordingly, increased Notch activity was sufficient to suppress tumorigenicity of aggressive PC3 prostate cancer cells.

Downstream of Notch activation, we showed that p21^WAF1/CIP1^ is a key target gene that mediates growth suppression even in prostate cancer cells with mutated and/or deleted p53. Smad3, previously reported to regulate p21^WAF1/CIP1^ expression and to functionally and/or biochemically interact with Notch [56], is itself a primary transcriptional target of Notch in prostate cells. Down-modulation of the Notch pathway in tumors does not correlate with histological grade of the lesions, suggesting that it may occur as a relatively early event, prior to the acquisition of a more malignant behavior. Consistently, in Tomlins [31] we found decreased expression of the Notch1 gene already in precursor lesions of prostate cancer (HGPIN). However, permanent down-modulation of the Notch pathway is also likely to be required for tumor development, as decreased Notch1 expression has been found also at late stages of cancer progression [30, 31]. In keeping with that, in our own dataset with a follow-up of more than 10 years, tumors associated to poor outcome were enriched in both Notch_high and in Notch_low samples (data not shown).

Previous studies showed that Notch1 is specifically expressed in myoepithelial cells of the basal layer of the prostate gland, while it is down-modulated in luminal cells [57]. Valdez and collaborators [58] discovered a positive feedback loop between stromal TGFβ and Notch signaling in basal cells, which appears to diminish prostate growth by limiting basal cell proliferation. In an experimental mouse model characterized by conditional deletion of the Notch1 gene, increased prostate cell proliferation and deranged differentiation with features resembling those of early neoplastic lesions [22] were found. The fact that the vast majority of prostate cancers usually display luminal as well as basal cell markers suggests that these cancers arise from stem cells that are at an early stage of commitment to differentiation [20, 21]. In this case, one would expect that aberrant or down-modulated Notch signaling is required for cancer development, an expectation supported by our findings.

In the present report we could also show that prostate cancer cells resistant to Notch1 growth inhibitory effects retain Notch-induced upregulation of pro-oncogenic genes also found in Notch_high tumors. This was the case of resistant PC3 clones, with high levels of Notch1 and of Notch1-induced targets: CXCL6, already found overexpressed in bone-tropic phenotypes where skeletal colonization is favored [51], and EPAS1, a hypoxia-inducible factor that promotes tumorigenesis, angiogensis, and metastasis by direct interaction with the 8q24 prostate cancer locus [52]. Our hypothesis is that, once such cells have escaped from p21-mediated Notch growth inhibition, they become more tumorigenic, in a context where many other important pathways are already deranged, thus favoring EMT and migration. In line with this, a previous study suggested a role for Notch1 signaling in the acquisition of an aggressive phenotype and Notch_high tumors were found enriched in both EMT and cell migration [59].

Taken together, our results can reconcile different and apparently contrasting results on the role of Notch1 in prostate cancer and suggest that Notch1 inhibition as therapy for this malignancy should be carefully contextualized by analyzing Notch1 expression in cancer cells and in their surrounding microenvironment.

## MATERIALS AND METHODS

### Cell culture, viruses and plasmids

By prostate epithelial cells (PrEC and hPEC) were obtained respectively from Cambrex and Dr M. Laiho (Helsinki, Finland) and maintained in PrEBM supplemented with bovine pituitary extract, epidermal growth factor, insulin, transferring, hydrocortisone, retinoic acid, epinephrine, triiodothyronine, and gentamicine-amphotericin solution (Cambrex). The short term cultured prostate cancer cell lines C1 IM to C45 IM were a gift of Dr A. Farsetti (Roma, Italy) and were isolated and cultured as described previously [60]. All other cancer cell lines were obtained from the ATCC and cultured as recommended by the ATCC. The plasmids rNERT-neo and neo were kindly provided by Dr U. Just (Christian-Albrechts-University of Kiel, Germany), the plasmids pSI-MSCV-CLPpuro-H1R-NotchRi6 and pSI-MSCV-CLPpuro-H1R-NotchRi7 (respectively sh-N1 n°6 and n°7, in the text) and control (sh-Ct) by Dr. T. Kiyono (National Cancer Center Research Institute, Tokyo) and the plasmid MSCV-neo-Notch1 and control by Dr C. Brisken (EPFL, Switzerland). The pRS-p21 n°1 and n°3 plasmids were respectively from Drs. R. Agami and R. Bernards (Dutch Cancer Research Institute), and the pRS-Smad3 plasmids from X-H Feng (Baylor College of Medicine). The pinco-Notch1 plasmid was obtained by inserting the cDNA of activated Notch1 (from digestion of the pcDNA3/hNIC by BamHI/XhoI) into the BamHI/EcoRI sites of the pinco-GFP vector [61]. Retroviruses were amplified in 293T packaging cells and the supernatant containing the viral particles was used to infect the cells during one hour together with polybren. Infection with the adenoviruses Ad-GFP, Ad-Notch1 and Adp53 was performed as previously described [62]. For transfection, cells were incubated in presence of a mixture of lipofectamine 2000 (Invitrogen) according to the manufacturer's protocol and either pcDNA3/hNIC, pcDNA3-flag-Smad3 or control plasmid. The plasmid expressing pcDNA3-flag-Smad3 was a gift of P. Ten Dijke (Leiden University Medical Center). For RNA transfection, cells were incubated in presence of a mixture of Interferin (Polyplus) according to the manufacturer's protocol, with the following siRNAs: sip63 n°1: 5′- GCACACAAUUGAAACGUACAGGCAA-3′, sip63 n°2: 5′-ACCAUGAGCUGAGCCGUGAAUUCAA-3′ or the siRNA control (Stealth siRNA, Thermo Fisher Scientific). 4-hydroxytamoxifen was purchased from Sigma and resuspended in 95% ethanol to make 1 mM stock solution, stored at −80°C.

### Prostate samples and laser capture microdissection (LCM)

Prostate cancer tissue samples containing at least 70% of neoplastic cells were obtained from 67 patients (age range: 50–75 years) who had undergone radical prostatectomy at the Hospital of Biella (Italy) and prostate biopsies were obtained from 10 healthy patients of the same age range, without any diagnosis of prostate tumor or BPH. All patients gave informed consent and the study was approved by the Ethical committee of Regione Piemonte. LCM was performed using an Arcturus XT microdissection system (Applied Biosystems, Carlsbad, CA, USA) as before [63].

### Alamar blue, clonogenicity and tumorigenicity assays

For Alamar Blue assay, cells were seeded at a density of 100 cells (for PC3 cells) or of 200 cells (for RWPE1 cells) per well of a 96 well plate in triplicate and cells density was further evaluated at the indicated days by incubating cells for two hours with the Alamar Blue Reagent according to the manufacturer's instructions (Biosource). Fluorescence excitation was performed at 544 nm and the emission was read at 590 nm on a fluorimeter (Molecular Devices). For cell survival assay, PC3 cells were transduced with a retroviral vector over-expressing Notch1 and GFP (MSCV-N1-GFP) or GFP alone (MSCV-GFP) as control. At days 2, 3, 6 and 9 after transduction, cells were trypsinized and % of GFP positive cells over total cells was determined by counting. For clonogenicity assay, cells were seeded at a density of 1000 cells (for PC3 cells) or 2000 cells (for RWPE1 cells) per 6-cm culture plates. Ten (for PC3 cells) or 13 (for RWPE1 cells) days later, cells were washed with PBS, fixed in 100% ethanol and stained in 0.1% crystal violet overnight before washing. For tumour formation in mice, cells were collected and 10^6^ cells were mixed with 70 μl of Matrigel (BD Biosciences) before subcutaneous injection in nude mice. Four weeks after, tumors were dissected, and analyzed by fluorescent microscopy.

### Quantitative real time PCR (RT-qPCR), ChIP assay and microarray hybridization

RT-qPCR and ChIP assays were performed as previously described [18] and the list of specific primers used for this study is provided in Supplementary Table S3.

For microarray hybridization of LNCaP and PC3 cell lines plus/minus Notch1, total RNA (5–8 μg) was used as template for double stranded cDNA preparations with T7-(dT)24 oligonucleotide primers for the first strand reaction. The resulting cDNAs were used for preparation of biotin-labeled cRNA probes preparation and hybridization to the Affymetrix U95 Av2 gene expression platform containing 12,626 probe sets according to manufacturer's recommendation (two chips per sample were generated, following manufacturer's instructions).

For prostate tissue microarray hybridization, prostate tumor (*n* = 67) and normal prostate (*n* = 10) tissues were co-hybridized with a commercial pool of RNA from organ donor healthy prostates (Becton Dickinson). Tissues were homogenized (Ultra – TurraxT8, Ika – Werke) and totRNA was isolated following TriReagent (Sigma Aldrich) protocol. mRNA was amplified by means of Amino Allyl MessageAmp I or II aRNA Kit (Ambion Inc.), according to manufacturer's protocol; only one round of amplification was performed. Labeling was performed using NHS ester Cy3 or Cy5 dies (Amersham, GE Healthcare) able to react with the modified aaRNA. The same quantity of differentially labeled sample and reference was put together, fragmented and hybridized to oligonucleotide glass array with sequences representing 27,958 Entrez Gene RNAs and 7,419 lincRNAs (Human Gene Expression 8 × 60K Microarray, Agilent Technologies). After the hybridization step, slides were washed following Agilent procedure and scanned with the dual-laser Agilent scanner G2505C. For each experiment, a dye-swap replicate was performed. totRNA and mRNA quality, quantity and labeling were checked by Agilent 2100 bioanalyzer and NanoDrop ND-1000 Spectrophotometer (Thermo Scientific).

### Microarray analysis

Raw Affymetrix CEL files were loaded into R and analyzed with RMA (Robust Multichip Average). The LIMMA (LInear Models for Microarray Analysis) package was then used to identify differentially expressed genes upon Notch1 activation. Data can be retrieved from GEO # GSE74631.

Agilent images were analyzed using the Feature Extraction software (Agilent Technologies). Raw data elaboration was carried out with Bioconductor (www.bioconductor.org) [64] using R statistical language. Background correction was performed with the normexp method with an offset of 50, loess was used for within-array normalization and A-quantile for between-array normalization. Data can be retrieved from GEO #GSE60329.

Additional publicly available gene expression array data were obtained from GEO (http://www.ncbi.nlm.nih.gov/geo/) and from TCGA (The Cancer Genome Atlas) Research Network. The Ambs' raw .CEL files were loaded into R and analyzed with RMA (Robust Multichip Average). For Taylor's dataset, processed data by the authors were used. TCGA data on prostate adenocarcinoma were analyzed with the edgeR package of Bioconductor.

The LIMMA (LInear Models for Microarray Analysis) package was then used to identify genes differentially expressed in Notch_low versus Notch_high tumors. The empirical Bayes method was used to compute a moderated t-statistics [65]. *p*-values were adjusted for multiple testing by using a false discovery rate (FDR) correction (Benjamini and Hochberg method).

### Images were analyzed using the feature extraction software (agilent technologies)

Raw data elaboration was carried out with Bioconductor (www.bioconductor.org) [64] using R statistical language. Background correction was performed with the normexp method with an offset of 50, loess was used for within-array normalization and A-quantile for between-array normalization. Data can be retrieved from GEO # GSE60329.

Additional publicly available gene expression array data were obtained from GEO (http://www.ncbi.nlm.nih.gov/geo/) and from TCGA (The Cancer Genome Atlas) Research Network. The Ambs' raw. CEL files were loaded into R and analyzed with RMA (Robust Multichip Average). For Taylor's dataset, processed data by the authors were used. TCGA data on prostate adenocarcinoma were analyzed with the edgeR package of Bioconductor.

The LIMMA (LInear Models for Microarray Analysis) package was then used to identify genes differentially expressed in Notch_low versus Notch_high tumors. The empirical Bayes method was used to compute a moderated t-statistics [65]. *p*-values were adjusted for multiple testing by using a false discovery rate (FDR) correction (Benjamini and Hochberg method).

### GO enrichment and network analysis

In order to look for any overrepresented biological processes (BP5) of the Gene Ontology (GO), we used the functional annotation tool available within DAVID Website (http://david.abcc.ncifcrf.gov/), using the lists of differentially expressed genes in prostate tumors versus healthy control samples.

MetaCore^TM^ version 6.23 (Thomson Reuters) was used for network analysis, that was applied to differentially expressed genes differentially expressed in LNCaP and PC3 cells upon acute Notch activation.

### Western blot

Total proteins were extracted as previously described [66]. Nuclear extracts were prepared as followed: cells were first lysed for 5 minutes in L1 buffer (50 mM Tris pH 8.0, 2 mM EDTA, 0.1% NP-40, 10% Glycerol) supplemented with proteases inhibitors. Nuclei were then pelleted at 3000 rpm in microfuge and resuspended in L2 buffer (50 mM Tris pH 8.0, 1% SDS, 5 mM EDTA). Immunodetection of Notch1, Flag, HES1, p21^WAF1/CIP1^, Smad3, TATA-box binding protein (TBP) and β-actin was performed using the primary antibodies sc-6014 (Santa-Cruz), F-3165 (Sigma), HES1 (gift of T. Sudo),sc-6246 (Santa-Cruz), 51–1500 (Zymed Laboratories), ab-818 (Abcam) and sc-1616 (Santa-Cruz) respectively.

## SUPPLEMENTARY MATERIALS FIGURES AND TABLES






