# Supplementary material for: Dual tumor suppressing and promoting function of Notch1 signaling in human prostate cancer
Source: Oncotarget. 2016 Jun 30;7(30):48011–26. doi: 10.18632/oncotarget.10333 (PMC5216996; doi:10.18632/oncotarget.10333)
Supplement: Supplementary file 1 [file oncotarget-07-48011-s001.pdf]

Dual tumor suppressing and promoting function of Notch1 signaling in human prostate cancer

Supplementary Materials

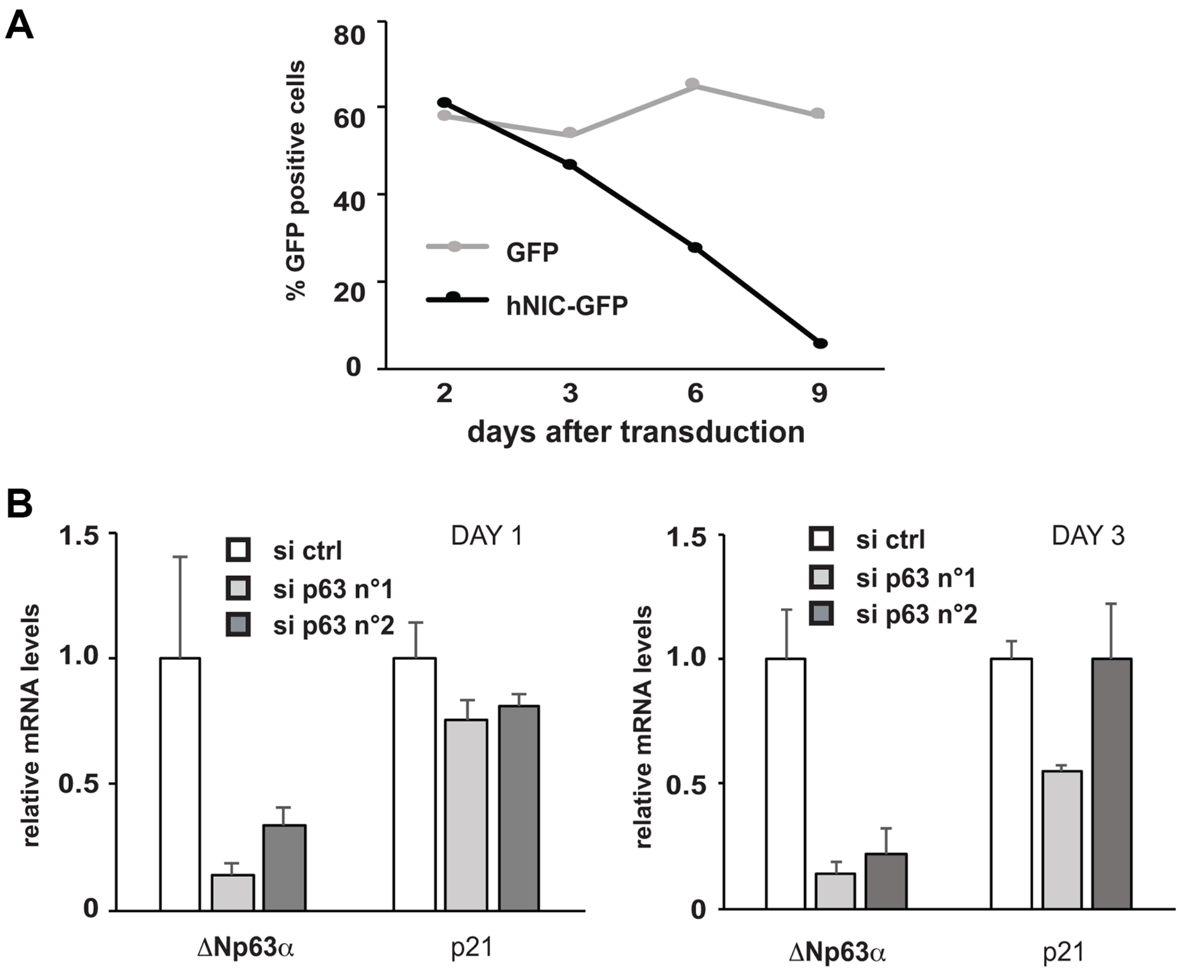

Supplementary Figure S1: Effects of Notch1 overexpression and p63 silencing respectively on cell growth and p21$^{WAF1/Cip1}$ expression in PC3 cells. (**A**) PC3 cells were infected with a retrovirus expressing the activated form of Notch 1 in parallel with GFP (hNIC-GFP) or a control virus expressing GFP alone (GFP). The percentage of retrovirally infected GFP-positive cells was determined by fluorescence microscopy at the indicated days until day 9. (**B**) PC3 cells were transfected with 2 different siRNA against DNp63α in parallel with control siRNA and RNA expression of DNp63α as well as of p21$^{WAF1/Cip1}$ was analyzed by real time RT-PCR (using 36β4 mRNA for normalization) 1 day (left panel) or 3 days after transfection (right panel).

Supplementary Table S1: Genes up-regulated by Notch1 in LNCaP and/or PC3.
See Supplementary_Table_S1

Supplementary Table S2: Genes that are induced or repressed by Notch1 in PC3 and/or LNCaP cells and that are concomitantly differentially expressed in prostate tumors according to Notch1 levels in at least 3 independent datasets. See Supplementary_Table_S2

Supplementary Table S3: Primers used for RT-QPCR reactions

Gene	Forward primer (5′-3′)	Reverse primer (5′-3′)
36β4	TCACTGTGCCAGCTCAGAAC	AATTTCAATGGTGCCTCTGG
18s	GCAATTATTCCCCATGAACG	GGCCTCACTAAACCATCCAA
β-actin	GGACTTCGAGCAAGAGATGG	AGGAAGGAAGGCTGGAAGAG
HEY1	TCTGAGCTGAGAAGGCTGGT	GCGCGTCAAAGTAACCTTTC
NOTCH 1	GAACCAATACAACCCTCTGC	AGCTCATCATCTGGGACAGG
HES1	GGTGCTGATAACAGCGGAAT	TGAGCAAGTGCTGAGGGTTT
P21WAF1/CIP1	CCCAAGCTCTACCTTCCCAC	ACAGGTCCACATGGTCTTCC
ΔNP63A	ATTGCATCACTGTATCATTTTCT	TGCTCTGTGGGGACCTTTCA
SMAD3	TGAGTTCGCCTTCAATATGAAGAA	CAGGAGGTAGAACTGGTGTCTCTACTCT
EPAS1	TTTGCAACTCCCTGGGTAAG	TAGTTGGCCCAATGTGCTTT
CXCL6	TTGAAGAGTGTGGGGGAAAG	CAATTGCCAAAGGGTTCAAT
