# Supplementary material for: Dual tumor suppressing and promoting function of Notch1 signaling in human prostate cancer
Source: Oncotarget. 2016 Jun 30;7(30):48011–26. doi: 10.18632/oncotarget.10333 (PMC5216996; doi:10.18632/oncotarget.10333)
Supplement: Supplementary file 3 [file oncotarget-07-48011-s003.docx]

**Supplemental Table 2:** Genes that are induced or repressed by Notch1 in PC3 and/or LNCaP cells and that are concomitantly differentially expressed in prostate tumors according to Notch1 levels in at least 3 independent datasets.

| **Genes up-regulated in Notch_high PCas and induced or repressed by Notch2 in PC3 and/or LNCaP cells** | |
| --- | --- |
| **Gene Symbol** | **Gene Name** |
| HES1 | hairy and enhancer of split 1, (Drosophila) |
| IGFBP4 | insulin-like growth factor binding protein 4 |
| ITGA3 | integrin, alpha 3 (antigen CD49C, alpha 3 subunit of VLA-3 receptor) |
| ROBO1 | roundabout, axon guidance receptor, homolog 1 (Drosophila) |
| ITGB4 | integrin, beta 4 |
| KRT15 | keratin 15 |
| MUC4 | mucin 4, cell surface associated |
| TNFAIP2 | tumor necrosis factor, alpha-induced protein |
| NOTCH3 | notch 3 |
| CAV2 | caveolin 2 |
| ISL1 | ISL LIM homeobox 1 |
| VWA5A | von Willebrand factor A domain containing 5A |
| ZFP36L1 | ZFP36 ring finger protein-like 1 |
| CAV1 | caveolin 1, caveolae protein, 22kDa |
| PLAU | plasminogen activator, urokinase |
| ANP32E | acidic (leucine-rich) nuclear phosphoprotein 32 family, member E |
| STOM | stomatin |
| SNAI2 | snail family zinc finger 2 |
| HEPH | hephaestin |
| AOX1 | aldehyde oxidase 1 |
| RND3 | Rho family GTPase 3 |
| IL18 | interleukin 18 (interferon-gamma-inducing factor) |
| OAT | ornithine aminotransferase |
| IER3 | immediate early response 3 |
| MAP3K8 | mitogen-activated protein kinase kinase kinase 8 |
| CXCL6 | chemokine (C-X-C motif) ligand 6 |
| GPR126 | G protein-coupled receptor 126 |
| ASPA | aspartoacylase |
| KLF5 | Kruppel-like factor 5 (intestinal) |
| DUSP2 | dual specificity phosphatase 2 |
| BHLHE40 | basic helix-loop-helix family, member e40 |
| EPAS1 | endothelial PAS domain protein 1 |
| ID1 | inhibitor of DNA binding 1, dominant negative helix-loop-helix protein |
| ARHGEF2 | Rho/Rac guanine nucleotide exchange factor (GEF) 2 |
| AHNAK2 | AHNAK nucleoprotein 2 |
| TNC | tenascin C |
|  |  |
| **Genes up-regulated in Notch_low PCas and induced or repressed by Notch2 in PC3 and/or LNCaP cells** | |
| **Gene Symbol** | **Gene Name** |
| GREB1 | growth regulation by estrogen in breast cancer 1 |
|  |  |
| ABCC4 | ATP-binding cassette, sub-family C (CFTR/MRP), member 4 |
| MIPEP | mitochondrial intermediate peptidase |
| HOXC6 | homeobox C6 |
| CBS | cystathionine-beta-synthase |
| CGREF1 | cell growth regulator with EF-hand domain 1 |
| TFF3 | trefoil factor 3 (intestinal) |
| CLGN | calmegin |
| TUBB2A | tubulin, beta 2A class IIa |
| PYCR1 | pyrroline-5-carboxylate reductase 1 |
| KLK3 | kallikrein-related peptidase 3 |
| HIST1H1C | histone cluster 1, H1c |
| GDF15 | growth differentiation factor 15 |
